# Lgr6: From Stemness to Cancer Progression

**Published:** 2019-02-05

**Authors:** Emanuela Cortesi, Juan-Jose Ventura

**Affiliations:** Translational Cell and Tissue Research, Dept. of Imaging and Pathology, KU Leuven, Belgium

## Abstract

Lung cancer is the leading cause of cancer-related deaths worldwide with poor prognosis, mainly due to the delay in the diagnosis. Adenocarcinoma, a subtype of non-small cell lung cancer, has the highest incidence and significant recurrence rates. Experimental and clinical researches suggested that the presence of cancer stem cells could support the development, malignization and resistance of lung cancer. Unfortunately, our knowledge in the field is still limited.

Here we report our findings regarding a cell population expressing LGR6, an epithelial stem cell marker. Under the pressure of a fine regulated p38α MAPK/mir-17-92 axis, LGR6^+^ stem cells produce differentiated bronchioalveolar cells, in the normal lung.

LGR6 is enriched in tumour cells during adenocarcinoma progression. Similar to normal stem cells, LGR6^+^ cancer cells show self-renewal and differentiation capacities, alongside with a higher oncogenic potential. Our studies suggest a disruption in the p38α MAPK/mir-17-92 network, that enhances Wnt pathway activity, could be responsible for the selection of malignant LGR6^+^ tumour cells. These results support the existence of a cell population with stem-like characteristics and strong oncogenic potential. This population could be useful for predictive diagnosis and a novel target for improved and more effective therapies against metastases and recurrences of lung adenocarcinomas.

## Introduction

Lung cancer (LC) is the most common type of cancer. In 2012, LC was estimated to be responsible for nearly 1.8 million deaths worldwide, almost 13% of all cancers in total^1^,^2^, with an overall death rate that exceeded the numbers of breast, colorectum and pancreatic cancers combined^3^. More than half of the newly diagnosed LC patients die within one year from the diagnosis, and the overall 5-year survival is lower than 18%^4^. Together with Small Cell LC (SCLC), Non-Small Cell LC (NSCLC) is one of the two main types of LC and accounts for approximately 85% of the total.

Among the three NSCLC subtypes, lung adenocarcinoma (ADC) is the most common^1^,^6^. Unfortunately, almost 40% of NSCLC patients are diagnosed at an advanced stage of the disease^5^ and, despite the available therapies, LC often trends to produce recurrences. Current knowledge in the field point to the existence of resident cell populations with stem-like properties as the origin of tumour recurrence and drug-resistance^7^,^8^. Similar to normal stem cells (SCs), cancer stem cells (CSCs) would retain self-renewing and multipotent capabilities and would be responsible for tumour development and maintenance^9^,^10^. Due to their quiescence, CSCs could also be resistant to drug treatments, that target proliferating cells, thus leading to an enrichment of cell populations that harbour the potential to generate more aggressive and metastatic tumours^11^.

Unfortunately, although several groups have extensively reported the existence of certain populations of putative SCs in mouse lungs^12^–^16^, our knowledge about lung SCs in humans is still poor^17^,^18^.

## LGR6+ lung SCs

In the last decade, many epithelial SC markers have been proposed in different epithelial tissues^19^. In the lung, the undisclosed hierarchical structure of the epithelial SCs still represents a handicap for the validation of biomarkers in order to identify and classify putative lung SCs and CSCs^20^.

The members of the family of leucine-rich repeat-containing G-protein-coupled Receptors (LGR) have been proposed as specific markers labelling epithelial SCs, with a special relevance of the LGR isoforms 4, 5 and 6^21^. This is a family of membrane proteins involved as regulators for several receptors and in particular it has been described as enhancers of Wnt signaling pathway^22^. Within the skin, LGR6 was shown to tag a SC population that can differentiate into all tissue-specific cell lineages, including those of the sebaceous gland, hair follicle and interfollicular dermis^23^. In a similar way, LGR6 has recently been proposed as a lung SCs marker^17^.

In the human lung, LGR6 positive (LGR6^+^) cells are a subpopulation located in the bronchioalveolar compartment. *In vitro*, LGR6^+^ cells showed ability to self-renewal, with unlimited expansion, and the expression of SCs markers (SOX9, LGR5-6, ITGA6). *In vivo* studies disclosed the potential of these cells to differentiate into several bronchioalveolar mature cell types (Club cells, alveolar type I and II cells), replacing the damaged tissue^17^. The regenerative process is allowed and regulated by a balanced cross-talk between stem and stromal cells of the lung^16^. When injected under the kidney capsule, LGR6^+^ SCs are able to recruit connective and endothelial cell components to the graft, generating a functional SC niche^17^.

A molecular network involving several cytokines and chemokines (SDF-1, TNFα, TGFβ) coordinates the recruitment of the stromal fibroblasts, that actively participate in the control of SCs differentiation. Within the niche, LGR6^+^ SCs closer to the fibroblasts remain quiescent and undifferentiated; however, LGR6^+^ cells that moved away from the niche are prompt to differentiate, producing a bronchioalveolar-like epithelium. The paracrine signals coming from the SCs induce the fibroblasts to release angiogenic molecules (VEGF, IL-8), that lead to the recruitment of the endothelial cells. These studies showed that p38α MAPK signalling pathway plays a central role in this fine-tuned cross-talk between stem and stromal cells.

p38α MAPK pathway is a major component in lung tissue homeostasis, collaborating in lung SCs fate decision^14^. Studies in mice have shown that the activity of this kinase is pivotal for the correct maturation of the alveolar epithelium. p38α induces lung differentiation transcription factors, including C/EBPα and GATA6, and downregulates several others involved in the proliferation of stem and progenitor cells^14^,^24^,^25^. The miR-17-92 cluster is a major mediator of this regulation and its activity is negatively controlled through the transcriptional activation of p53 by the p38α MAPK pathway^17^. On the other side, miR-19, a member of the miR-17-92 family, targets and knocks-down p38α protein levels^14^.

Also known as oncomiR-1, the miRNA-17-92 cluster is a pool of oncogenic microRNAs that has been found to be overexpressed in several tumours, including LC. As previously reported, p38α deficiency, or p53 inactivating mutations, lead to an increase in miR17-92 levels (e.g. mir-19), downregulating lung-specific transcription factors and generating cells with higher proliferative capacities^26^. Disruption in the balance of this network was observed in LGR6^+^ cancer cells, supporting the SC-like properties of these lung tumour cells.

Although p38α is not considered an oncogene and it is usually not present in gene expression cancer-related databases, we found in the past an increase in the protein levels of this kinase in human lung tumors. Thus, this data suggested that p38α may play a role as adjuvant for specific cancers, contributing to their initiation and/or progression^11^.

## LGR6+ SCs in Human Lung ADC

After the analyses of > 50 NSCLCs, our group has reported new findings regarding the existence of LGR6^+^ cells in human lung ADCs. Although LGR6 expression was observed in the tumour burden in earlier (I and II) and later (III, IV and metastatic) stages, a stage-related enrichment in LGR6^+^ cells was found during cancer development.

In order to assess their stem-like identity and the tumorigenic potential, the molecular profile of sorted LGR6^+^ tumour cells, from different stages of human lung ADC, was analysed and compared to LGR6^-^ cancer cells. In both cell populations, lung markers were expressed at similar levels (SP-C, CC-10, MUC5AC) and only the marker AQP5, a known a marker for LC malignancy, was overexpressed in LGR6^+^ cells, that showed higher tumorigenic and self-renewal potential. The *in vitro* ability of these cells to produce visible macroscopic colonies was therefore confirmed by angiogenic molecules (VEGF, IL-8), that lead to the *in vivo* studies (LGR6^+^ cells at stage II), using xenografting recruitment of the endothelial cells. These studies showed that p38α MAPK signalling pathway plays a central role in this fine-tuned cross-talk between stem and stromal cells. experiments in immunodeficient mice. After intravenous injection, newly formed lung tumours arising from human LGR6^+^, but not from LGR6^-^ cells were observed. The detected tumour burdens expressed lung-specific markers, including SP-C and CC- 10, and showed only partially LGR6 expression.

One of the proposed hallmarks of CSCs is a larger potential to produce metastasis^27^. This ability was confirmed in LGR6^+^ CSCs, that showed stronger expression of stemness (LGR6) and loss of epithelial (E-Cadherin) markers, from early to late stages. In addition, although the expression of alveolar and bronchiolar markers was present in stage I and II ADCs, only marginal expression of SP-C and CC-10 was observed in advanced-stage tumours.

Previous findings regarding the p38α MAPK/miR-17-92 cross-talk were endorsed in ADC. Molecular analysis of the cluster members (mir-17, mir-18, mir-19a, mir-19b, mir-20 and mir-92) confirmed an increased gene expression, during tumour progression. miR-17-92 cluster overexpression was linked to the transcriptional repression of p53, confirmed by strong reduction of p21 mRNA levels. In addition, we found that the reduced protein levels of p38α in late-stage ADC could be accounted to a novel negative-feedback loop with miR-19 that specifically targets the 3’-UTR of p38α.

As in other tissues, in the lung, Wnt signaling is one of the key pathways involved in the regulation of development, SCs maintenance in adults and as well as in tumorigenesis^28^–^30^. In the β-catenin dependent (canonical) Wnt pathway, when WNT binds to the FZD receptor, it activates a downstream cascade that leads to the inhibitory phosphorylation of Glycogen Synthase Kinase 3-beta (GSK-3β), allowing the nuclear shuttling of β-catenin^30^. The translocated β-catenin arranges a complex with members of the TCF/LEF family of transcription factors, resulting in an enhanced expression of Wnt target genes. During lung ADC progression, increased phosphorylation of GSK-3β and massive β-catenin nuclear translocation were observed. At advanced stages, further analyses of the molecular profile of these cells confirmed elevated mRNA levels of the Wnt pathway enhancer LRP6 and specific targets, such as LEF1, TCF4 and AXIN2, as well as a diminished expression of Wnt repressors, including WIF1, GREMLIN, DKK1 and DAB2.

As previously mentioned, LGR6 is a promoter of Wnt receptor signalling, through its interaction with LRP6, amplifying the activation of the pathway. We observed that this takes place in a stage-dependent manner, during tumour progression. Further studies showed that, in LGR6^+^ cells, p38α deficiency results in an increased inhibition of GSK-3β, that is coupled to higher expression of Wnt target specific genes, suggesting that p38α reduction mediates the enhancement Wnt signalling, during ADC progression, thus promoting the expansion and selection of LGR6^+^ CSCs.

All these analyses confirmed an existing correlation between the lack of the cellular differentiation, the increased expression of Wnt signalling components and p38α deficiency in LGR6^+^ cancer cells. From early to late stages, loss of p38α enables the selection of LGR6^+^ SCs, however still maintaining their self-renewal potential. These changes lead to the formation of tumours with poorly differentiated cell components and high metastatic potential.

## Conclusion and future perspectives

In this work, we have unveiled a cellular network (miR-17-92/p38α) with a putative adjuvant role in lung ADC. This network plays a distinct role in the regulation of a lung SC population (LGR6^+^), guiding its differentiation and self-renewal. Disruption of the balance between miR-17-92 cluster and p38α MAPK is more relevant in LGR6^+^ cells, as it modulates the activity of Wnt signal. These cells are benefited by Wnt enhancement, which promotes a positive selection. As LGR6^+^ cells present SC features, unbalanced miR-17-92/p38α means lack of differentiation while stronger self-renewal. As we have shown before, another property of LGR6^+^ SCs is being able to colonize and expand in different conditions and tissues^17^. These findings contribute to better understand how lung ADC progression leads to more aggressive cancers and which cell population might be behind the recurrence and resistance to LC therapies. Our work provides new potential cellular and molecular targets that can be used in the earlier detection, prediction and treatment of LC.

## References

[1] Reck M, Rabe KF (2017). Precision Diagnosis and Treatment for Advanced Non–Small-Cell Lung Cancer. N Engl J Med.

[2] Ferlay J, Soerjomataram I, Dikshit R (2015). Cancer incidence and mortality worldwide: Sources, methods and major patterns in GLOBOCAN 2012. Int J Cancer.

[3] American Cancer Society (2015). Cancer Facts and Figures.

[4] Available online: http://www.cancer.org/acs/groups/content/@editorial/documents/document/acspc-044552.pdf

[5] National Cancer Institute SEER Cancer Statistics Review, 1975-2011.

[6] Available online: http://seer.cancer.gov/csr/1975_2011/

[7] Stahel R, Peters S, Baas P (2013). Strategies for improving outcomes in NSCLC: a look to the future. Lung Cancer.

[8] Denisenko TV, Budkevich IN, Zhivotovsky B (2018). Cell death-based treatment of lung adenocarcinoma article. Cell Death Dis.

[9] Al-Hajj M, Wicha MS, Benito-Hernandez A (2003). Prospective identification of tumorigenic breast cancer cells. Proc Natl Acad Sci U S A.

[10] Cho RW, Wang X, Diehn M (2008). Isolation and molecular characterization of cancer stem cells in MMTV-Wnt-1 murine breast tumors. Stem Cells.

[11] Gupta PB, Chaffer CL, Weinberg RA (2009). Cancer stem cells: mirage or reality?. Nat Med.

[12] Lundin A, Driscoll B (2013). Lung cancer stem cells: progress and prospects. Cancer Lett.

[13] Hardavella G, George R, Sethi T (2016). Lungcancer stem cells – characteristics, phenotype. Transl Lung Cancer Res.

[14] Giangreco A, Reynolds SD, Stripp BR (2002). Terminal Bronchioles Harbor a Unique Airway Stem Cell Population That Localizes to the Bronchoalveolar Duct Junction. Am J Pathol.

[15] Kim CF, Jackson EL, Woolfenden AE (2005). Identification of bronchioalveolar stem cells in normal lung and lung cancer. Cell.

[16] Ventura JJ, Tenbaum S, Perdiguero E (2007). p38a MAP kinase is essential in lung stem and progenitor cell. Nat Genet.

[17] Teisanu RM, Lagasse E, Whitesides JF (2008). Prospective isolation of bronchiolar stem cells based upon immunophenotypic and autofluorescence characteristics. Stem Cells.

[18] Chapman HA, Li X, Alexander JP (2011). Integrin alpha6beta4 identifies an adult distal lung epithelial population with regenerative potential in mice. J Clin Invest.

[19] Oeztuerk-Winder F, Guinot A, Ochalek A (2012). Regulation of human lung alveolar multipotent cells by a novel p38a MAPK/miR-17-92 axis. EMBO J.

[20] Eramo A, Lotti F, Sette G (2008). Identification and expansion of the tumorigenic lung cancer stem cell population. Cell Death Differ.

[21] Kumar PA, Hu Y, Yamamoto Y (2011). Distal airway stem cells yield alveoli in vitro and during lung regeneration following H1N1 influenza infection. Cell.

[22] Templeton AK, Miyamoto S, Babu A (2014). Cancer stem cells: progress and challenges in lung cancer. Stem Cell Investig.

[23] Barker N, van Es JH, Kuipers J (2007). Identification of stem cells in small intestine and colon by marker gene Lgr5. Nature.

[24] Reya T, Clevers H (2005). Wnt signalling in stem cells and cancer. Nature.

[25] Snippert HJ, Haegebarth A, Kasper M (2010). Lgr6 marks stem cells in the hair follicle that generate all cell lineages of the skin. Science.

[26] Hui L, Bakiri L, Mairhorfer A (2007). p38alpha suppresses normal and cancer cell proliferation by antagonizing the JNK-c-Jun pathway. Nat Genet.

[27] Liu Y, Martinez L, Ebine K (2008). Role for mitogen-activated protein kinase p38 alpha in lung epithelial branching morphogenesis. Dev Biol.

[28] Mogilyansky E, Rigoutsous I (2013). The miR-17/92 cluster: a comprehensive update on its genomics, genetics, functions and increasingly important and numerous roles in health and disease. Cell Death and Diff.

[29] de Lau W, Barker N, Low TY (2011). Lgr5 homologues associate with Wnt receptors and mediate R-spondin signalling. Nature.

[30] Mucenski ML, Wert SE, Nation JM (2003). Beta-catenin is required for specification of proximal/distal cell fate during lung morphogenesis. J Biol Chem.

[31] Nusse R (2008). WNT signaling and stem cell control. CSH Symp Quant Biol.

[32] Zhan T (2017). Wnt signaling in cancer. Oncogene.

